# Reading the Leaves’ Palm: Leaf Traits and Herbivory along the Microclimatic Gradient of Forest Layers

**DOI:** 10.1371/journal.pone.0169741

**Published:** 2017-01-18

**Authors:** Stephanie Stiegel, Martin H. Entling, Jasmin Mantilla-Contreras

**Affiliations:** 1 University of Hildesheim, Institute of Biology and Chemistry, Ecology and Environmental Education Group, Hildesheim, Germany; 2 University of Koblenz-Landau, Department of Ecosystem Analysis, Landau, Germany; University of Illinois at Chicago, UNITED STATES

## Abstract

Microclimate in different positions on a host plant has strong direct effects on herbivores. But little is known about indirect effects due to changes of leaf traits. We hypothesized that herbivory increases from upper canopy to lower canopy and understory due to a combination of direct and indirect pathways. Furthermore, we hypothesized that herbivory in the understory differs between tree species in accordance with their leaf traits. We investigated herbivory by leaf chewing insects along the vertical gradient of mixed deciduous forest stands on the broad-leaved tree species *Fagus sylvatica* L. (European beech) with study sites located along a 140 km long transect. Additionally, we studied juvenile *Acer pseudoplatanus* L. (sycamore maple) and *Carpinus betulus* L. (hornbeam) individuals within the understory as a reference of leaf traits in the same microclimate. Lowest levels of herbivory were observed in upper canopies, where temperatures were highest. Temperature was the best predictor for insect herbivory across forest layers in our study. However, the direction was opposite to the generally known positive relationship. Herbivory also varied between the three tree species with lowest levels for *F*. *sylvatica*. Leaf carbon content was highest for *F*. *sylvatica* and probably indicates higher amounts of phenolic defense compounds. We conclude that the effect of temperature must have been indirect, whereby the expected higher herbivory was suppressed due to unfavorable leaf traits (lower nitrogen content, higher toughness and carbon content) of upper canopy leaves compared to the understory.

## Introduction

Insect herbivores play an important role in ecosystems and affect their structure and function [1,2]. Leaf area loss to insects reduces tree growth [3,4] and redirects primary production into the herbivore food chain [5,6], altering material flows from canopies to forest soils [7,8]. These interactions are influenced by the environment, essentially by climatic conditions (temperature and air humidity). Effects of climate on insect herbivory are complex because any factor can affect the insect and the plant at the same time, sometimes with opposite consequences for herbivory.

For example, temperature can affect insect physiology and behavior directly or indirectly through climate-induced changes of host plants [9]. Temperature determines herbivore growth and development [10,11], movement or activity rates [12], and distribution [13,14] and therefore influences feeding intensity.

Herbivore insect developmental rates increase with temperature. In contrast, limited and inconsistent effects are found for direct effects of humidity [15]. In contrast to temperature, under a wide range of humidity conditions there is no optimal level and herbivore insects can buffer humidity fluctuations [16]. Low levels of humidity leading to water stress of host plants can benefit defoliators through increased nitrogen content in plant tissue as an indirect effect [17]. Nonetheless, a meta-analysis revealed inconsistent responses of plant water stress for leaf chewers and miners [18].

Indirect effects of microclimate on herbivory can also be mediated by morphological and functional leaf traits like toughness, nutrients or defense compounds [19]. Carbon and nitrogen content of leaves are important predictors for herbivory levels [20]. Herbivory increases with leaf nitrogen, which has been linked to increased insect density, shorter development time, higher survival rates, and higher fecundity [21–23]. Carbon content is negatively correlated to leaf palatability [24] because mechanical or chemical defenses are often carbon-based [25,26]. Also leaf toughness negatively influences palatability for herbivore insects [27,28]. Negative changes of leaf traits for herbivore insects can suppress expected high rates of herbivory in warm environments [29]. Therefore, it is essential for research to consider the indirect effects of leaf traits besides direct effects of microclimate.

On a local scale, microclimate gradients are found across the different strata of forests. Abiotic factors change between layers within forest stands due to the vertical micro-environmental gradient [30,31]. Along the vertical gradient, microclimate is affected by the light regime, with increasing temperatures and decreasing humidity from understory to outer canopies, especially during sunshine. Outer canopies of trees experience high irradiances, vapor pressure deficits, and temperature fluctuations [32]. The understory of forests is characterized by low light, damped temperature fluctuations and generally high air humidity. Existing studies have evidenced variable patterns of herbivory for sun and shade leaves. Sun leaves of slow-growing species are either less attractive for herbivore insects [33,34] or reveal higher herbivory [35–38]. Under standardized temperatures, leaves from the outer canopy show higher palatability for herbivores [39] or no consistent difference compared to leaves from lower forest layers [40]. In contrast, under field conditions lower herbivory is observed on outer canopy leaves of *Fagus crenata* [41]. Few studies have focused on effects of leaf spatial location within tree canopies on herbivore insects. They reveal preference of shade leaves close to ground level for grazing [42], higher aggregation and feeding of beetles in upper canopies [43], and that difference in herbivory of upper and lower canopies varies in direction and magnitude depending on tree species [40]. This complicates generalizations about the responses of herbivore insects and patterns of herbivory to different microclimates on the same host plant.

Few studies include the whole vertical forest gradient. They show migration of moth larvae from canopies to understory seedlings due to changes in leaf quality [44] and higher herbivore performance in upper canopies with leaves containing more total nitrogen [39]. In this study, we investigated levels of herbivory caused by leaf chewing insects (leaf damage as percentage of missing leaf area) along the whole vertical gradient of forest stands on the broad-leaved tree species *Fagus sylvatica* L. (European beech). We compared leaf damage between different microclimates (understory, lower and upper canopy) of mature *F*. *sylvatica* individuals. Patterns of herbivory were analyzed with respect to abiotic factors (microclimate, leaf toughness and leaf nutrients) determining interactions and main predicting parameters for herbivory levels along the vertical forest gradient. Additionally, we studied juvenile *Acer pseudoplatanus* L. (sycamore maple) and *Carpinus betulus* L. (hornbeam) individuals within the understory as a reference of leaf traits in the same microclimate. We tested the following alternative hypotheses for the vertical forest gradient: 1) herbivory increases towards upper canopies of *F*. *sylvatica* through higher temperatures (direct effect of microclimate) or 2) oppositely, herbivory may be decreased in upper canopies through a shift of leaf traits towards lower palatability (indirect effect of microclimate with low leaf nitrogen content as well as high leaf carbon content and leaf toughness). Furthermore, we hypothesized that 3) herbivory in the understory differs between tree species in accordance with their leaf traits, i.e. increases with leaf nitrogen content and decreases with leaf carbon content and leaf toughness.

## Materials and Methods

### Ethics statement

Field work permits were issued by the responsible forestry offices Leinefelde (Winkelberg and Hubenberg), Neuhaus (Tiefentals Ebene), Münden (Klingenberg/Vaaker Berg), Wehrtal (Schieferstein), Hessisch-Lichtenau (Heiligenberg), Reinhausen (Bocksbühl), Michael Wienrich (Feuerkuppe), and Oldisleben (Heidelberg and Eichleite). The study sites comprise state forests and private forest. During this study no species that are protected by European or national laws were sampled. JMC received financial support from the University of Hildesheim. The funders had no role in study design, data collection and analysis, decision to publish, or preparation of the manuscript.

### Study area

**Fig 1:**
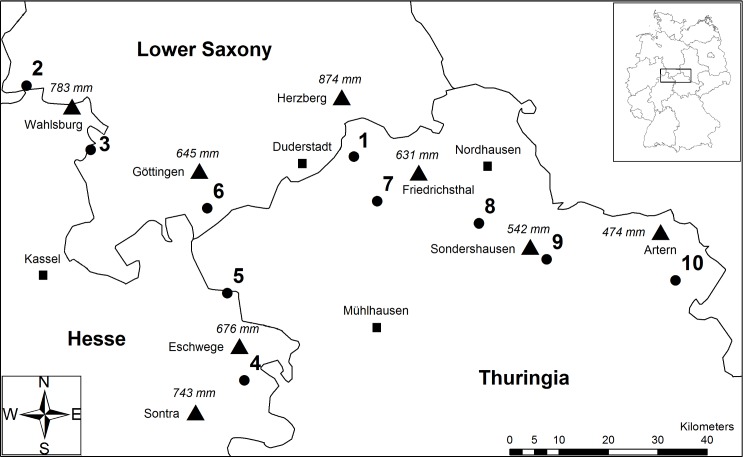
Overview of the ten forest study sites (circles) in Thuringia, Lower Saxony and Hesse. Weather stations (triangles) present mean annual precipitation (italic values) from reference period 1961–90 (German Weather Service). Forest sites: 1) Winkelberg, 2) Tiefentals Ebene, 3) Klingenberg/Vaaker Berg, 4) Schieferstein, 5) Heiligenberg, 6) Bocksbühl, 7) Hubenberg, 8) Feuerkuppe, 9) Heidelberg, and 10) Eichleite. Reprinted from BKG under a CC BY license, with permission from Bundesamt für Kartographie und Geodäsie, original copyright GeoBasis-DE / BKG 2015 (data changed).

The study was conducted in the centre of the distribution range of *F*. *sylvatica* within the hill and mountain region of Central Germany. We selected ten study sites with mixed deciduous forest stands in Thuringia, Lower Saxony and Hesse at elevations between 140 and 444 m.a.s.l. (Fig 1). Study sites were located along a 140 km long transect with increasing annual precipitation from east to west. According to the German Weather Service, mean annual precipitation ranges from 474 mm (Artern, Thuringia) to 874 mm (Herzberg, Lower Saxony) based on the reference period from 1961–1990 (Fig 1). Mean annual temperature in the study area was similar among the different forest sites and has increased from about 8°C to 9°C until the beginning of the 21^th^ century (S1 Table).

Most study sites occurred on the same geological substrate (lower Trias sandstone), with exceptions of Bocksbühl (upper Trias sandstone), Feuerkuppe, and Heidelberg (middle Triassic limestone). Our stand selection criteria were (i) closed canopy without major gaps, (ii) no significant presence of coniferous tree species, and (iii) stem circumference of adult beech individuals >1 m (average circumference: 1.80 m).

### Sampling trees

We selected most common tree species found in forest stands of the study area. Within the study area, *F*. *sylvatica* was the dominant broad-leaved tree species in mixed deciduous forests and therefore chosen as main object of focus. *Acer pseudoplatanus* and *C*. *betulus* were frequent in the understory at nine forest sites and included into the study to investigate differences and influences of leaf traits within the same forest layer. Within the ten forest sites, we undertook a random selection of juvenile and adult study tree individuals with north as well as south exposition. At each of the 20 exposition sites, three juvenile individuals (if available) of *F*. *sylvatica*, *A*. *pseudoplatanus* and *C*. *betulus* were selected in the understory for further analysis (S1 Fig). Additionally, we surveyed three adult individuals of *F*. *sylvatica* at the lower and upper canopy at each sample site. Selection of sample individuals at north and south exposition and in different forest layers represented a variation of microclimatic conditions. We selected a total of 60 adult and juvenile *F*. *sylvatica*, 41 juvenile *A*. *pseudoplatanus*, and 27 juvenile *C*. *betulus* individuals.

### Data collection

Microclimatic data (air temperature and relative air humidity) were measured every hour with data loggers (iButton, Model DS1923, Maxim Integrated) for two months (Jul-Aug 2012). Data loggers were installed within the understory (about 1 m height), as well as in lower (about 18 m height) and upper canopies (about 35 m height) of adult beech trees per sample site. We accessed the (inner) lower and (outer) upper canopy of adult beech trees by rope climbing. Foliage material was collected in June 2012 at each sample site and analyzed according to LEDA trait standards [45]. Single foliage samples of all available individuals were randomly taken within the layers of selected trees for analysis of SLA as indicator for toughness, nutrients (carbon and nitrogen), and herbivory (samples of twigs). All collected material was deep frozen until analyses were carried out. At some sample sites, juvenile tree individuals of *A*. *pseudoplatanus* and *C*. *betulus* were too small for harvesting enough foliage sample material. In this case, one sample consisted of several individuals. Given that chlorophyll content correlates with nitrogen content [46] measures of chlorophyll content index (CCI) were estimated with a CCM-200 plus Chlorophyll Content Meter (Opti-Sciences Inc., Hudson, USA). For each tree individual, we took ten values directly in the field in June 2012.

### Analyses

Microclimatic data of the three forest layers (understory, lower and upper canopy) were used as average daily values (6 am to 9 pm) from 01-Jul-2012 to 31-Aug-2012, based on the higher variation of temperature and humidity during day and its influence on plant-insect interactions. Specific leaf area was calculated for ten leaves of each tree individual (five leaves for *A*. *pseudoplatanus*). We scanned all fresh leaves with a flat-bed scanner and analyzed their areas with the computer image analysis system WinFOLIA (Régent Instruments Inc., Quebec, Canada). Then, we dried (70°C, 48 h) and weighed foliage samples.

We conducted nutritional analyses with mixed samples consisting of 10 (*F*. *sylvatica* and *C*. *betulus*) or 5 (*A*. *pseudoplatanus*) leaves per individual. Grounded samples were analyzed for total carbon and nitrogen content with a C/N elemental analyzer (Department of Plant Ecology and Ecosystem Research, University of Göttingen). As a combination of positive indicator for nutrients and negative indicator for defense compounds we used CN ratio. Specific leaf area, nutrient and chlorophyll parameters were used as mean values for each species and forest layer at the 20 sampled exposition sites.

We determined herbivory as the percent area of missing leaf tissue. Therefore, we scanned leaves of collected twigs with a flat-bed scanner (n(*F*. *sylvatica* understory) = 1801, n(*F*. *sylvatica* lower canopy) = 1120, n(*F*. *sylvatica* upper canopy) = 1425, n(*A*. *pseudoplatanus*) = 528, n(*C*. *betulus*) = 628) and then analyzed them with the computer image analysis system WinFOLIA (Régent Instruments Inc., Quebec, Canada). Leaf damage, including area of missing leaf edges, along the forest layer gradient was calculated for all species at the 20 exposition sites with the potential leaf size (existing plus missing leaf area) and the missing leaf area. Most leaf chewing insect species that feed on leaves in temperate broadleaved forests are polyphagous [47,48]. Based on the feeding traces on *F*. *sylvatica* some herbivore insect species that cause loss of leaf tissue were identified, all of which are polyphagus (*Diurnea fagella* D. & S., *Orchestes fagi* L., and *Phyllobius argentatus* L.).

For significant comparison of measured parameters and leaf damage along the vertical forest gradient and the three tree species in the understory, we performed statistical analyses in R (R development core team 2013, Version 3.0.2). Normal distribution for temperature, air humidity, SLA, leaf nitrogen and carbon content, C/N ratio, and chlorophyll content was assessed with Shapiro-Wilk-test and further tests were performed with ANOVA or Kruskal-Wallis and post-hoc-tests, respectively.

Determining parameters for herbivory were tested with generalized linear mixed models. For herbivory of *F*. *sylvatica*, model comparison was conducted for effects of forest layer, microclimate (2) and leaf traits (5) with the following model specification:

lmer(herbivory∼forestlayer+temperature+airhumidity+nitrogencontent+carboncontent+chlorophyll+CNratio+SLA+(1|site),REML=FALSE)

Model comparison for the three tree species in the understory was assessed for effects of species, microclimate (2) and leaf traits (5) with the following model specification:

lmer(herbivory∼species+temperature+airhumidity+nitrogencontent+carboncontent+chlorophyll+CNratio+SLA+(1|site),REML=FALSE)

Herbivory was square-root transformed and all models contained study site as a random effect. Calculations were done using the R libraries lme4 [49] and MuMIn [50]. We selected the best model based on the Bayesian Information Criterion (BIC). The lowest BIC value implied either fewer explanatory variables, better fit, or both combined. Linear regressions for herbivory and the best determining parameter were calculated with the following command:

lm(herbivory∼parameter)

## Results

### Microclimate

**Fig 2:**
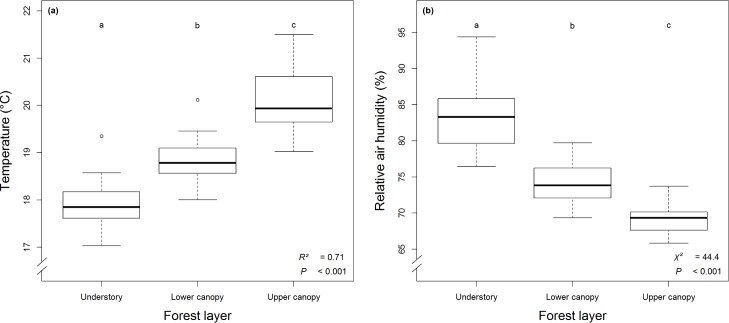
Microclimate along the vertical forest gradient. Microclimatic conditions for understory (n = 20), lower (n = 20) and upper canopy (n = 17) represented by (a) temperature and (b) relative air humidity. Boxplots with lowercase letters indicate significant differences using (a) ANOVA and Tukey's HSD (*P* < 0.05; df = 2) and (b) Kruskal-Wallis and post-hoc test (*P* < 0.05; df = 2).

Neither temperature nor relative air humidity showed significant differences between north (e.g. understory: 17.8°C ± 0.2 SD, 84.5% ± 3.3 SD) and south expositions (e.g. understory: 18.1°C ± 0.6 SD, 81.8% ± 5.3 SD). Thus, we excluded exposition as a parameter from further analyses. In contrast, microclimatic conditions varied significantly between the three forest layers across all sample sites. Average temperature increased from understory (17.9°C ± 0.5 SD) to lower canopy (18.8°C ± 0.5 SD) and upper canopy (20.1°C ± 0.8 SD) (Fig 2A). Air humidity and temperature showed a strong negative correlation (rho = -0.87, *P* < 0.001) (S1 Appendix). Accordingly, average air humidity decreased from understory (83.1% ± 4.5 SD) to lower canopy (74.1% ± 2.8 SD) and upper canopy (69.2% ± 2.2 SD) (Fig 2B). Therefore, forest layers represented different microclimates and were further investigated.

### Leaf parameters

**Fig 3:**
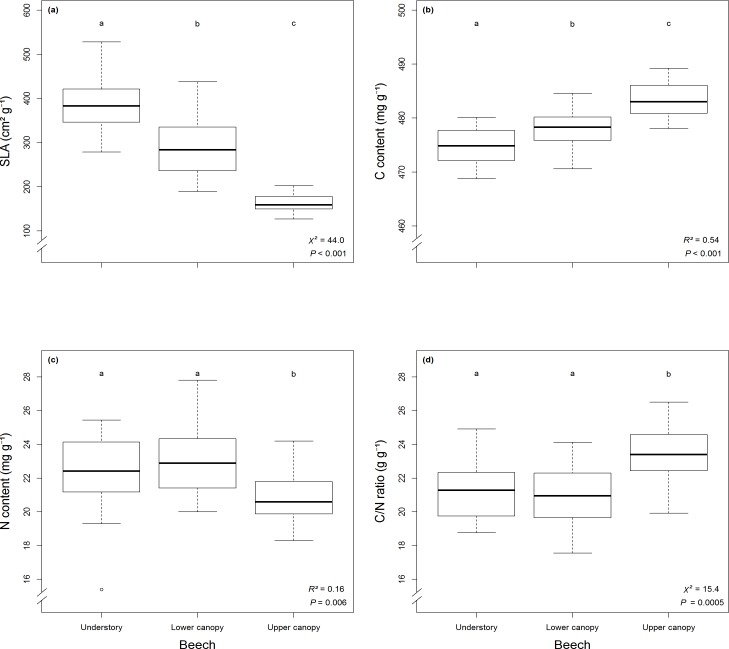
Leaf parameters for *F*. *sylvatica* (n = 60). Represented by (a) specific leaf area, (b) total carbon content, (c) total nitrogen content and (d) C/N ratio along forest layers. Boxplots with lowercase letters indicate significant differences using (b)-(c) ANOVA and Tukey's HSD (*P* < 0.05; df = 2) and (a) and (d) Kruskal-Wallis and post-hoc test (*P* < 0.05; df = 2).

Specific leaf area of *F*. *sylvatica* differed significantly between forest layers and decreased from understory to lower and upper canopy (Fig 3A). Leaves of upper canopies were smaller and thicker than in the understory. Total leaf carbon content increased on average from 474 mg g^-1^ ± 4 SD to 484 mg g^-1^ ± 5 SD along the forest layer gradient (Fig 3B). Average total leaf nitrogen content was significantly reduced in upper canopies (20.9 mg g^-1^ ± 2.1 SD) compared to lower canopies (23.1 mg g^-1^ ± 2.9 SD) and understory (22.4 mg g^-1^ ± 2.8 SD) (Fig 3C). Patterns of carbon and nitrogen content resulted in significantly augmented C/N ratio in upper canopies (23.3 g g^-1^ ± 2.3 SD) versus lower canopies (21.0 g g^-1^ ± 2.4 SD) and understory (21.6 g g^-1^ ± 3.0 SD) (Fig 3D). Values for chlorophyll content did not vary significantly along the vertical forest gradient and ranged on average between 13.2 CCI ± 1.8 SD (upper canopy), 13.4 CCI ± 1.7 SD (understory), and 14.2 CCI ± 2.2 SD (lower canopy).

Leaf traits were strongly correlated to microclimate (S1 Appendix). With increasing temperature, carbon content increased (rho = 0.59, *P* < 0.001) and SLA decreased (rho = -0.78, *P* < 0.001) resulting in a converse correlation pattern for air humidity with carbon content (rho = -0.65, *P* < 0.001) and SLA (rho = 0.89, *P* < 0.001). Further correlations were found between nitrogen content and SLA (rho = 0.55, *P* < 0.001), C/N ratio and SLA (rho = -0.60, *P* < 0.001) as well as nitrogen and chlorophyll content (rho = 0.26, *P* < 0.0429).

Within the same microclimate, leaf parameters were less variable between *F*. *sylvatica*, *A*. *pseudoplatanus* and *C*. *betulus* in the understory. Average values for SLA did not vary significantly due to high standard deviations (Table 1). Carbon content of *F*. *sylvatica* leaves was significantly higher than for leaves of *A*. *pseudoplatanus* and *C*. *betulus*. Other leaf parameters like nitrogen content, C/N ratio and chlorophyll content did not vary significantly between the three species in the understory. Chlorophyll content of *A*. *pseudoplatanus* leaves was on average highest but with higher variation of values (Table 1).

**Table 1:** Leaf parameters for juvenile *F*. *sylvatica* (n = 20), *A*. *pseudoplatanus* (n = 14), and *C*. *betulus* (n = 10) individuals represented by SLA, carbon and nitrogen content, C/N ratio and chlorophyll content in the understory.

Species	SLA(cm^2^ g^-1^)	C(mg g^-1^)	N(mg g^-1^)	C/N ratio(g g^-1^)	Chlorophyll(CCI)
***Fagus sylvatica***	388 ± 64^a^	474 ± 4^a^	22.4 ± 2.8^a^	21.6 ± 3.0^a^	13.4 ± 1.7^a^
***Acer pseudoplatanus***	345 ± 52^a^	456 ± 5^b^	23.0 ± 4.3^a^	20.6 ± 4.1^a^	15.1 ± 4.0^a^
***Carpinus betulus***	353 ± 35^a^	457 ± 6^b^	22.4 ± 2.8^a^	20.7 ± 2.5^a^	13.4 ± 2.4^a^

Mean values and standard deviation with lowercase letters indicate significant differences using Kruskal-Wallis and post-hoc test (*P* < 0.05; df = 2).

### Herbivory patterns

**Fig 4:**
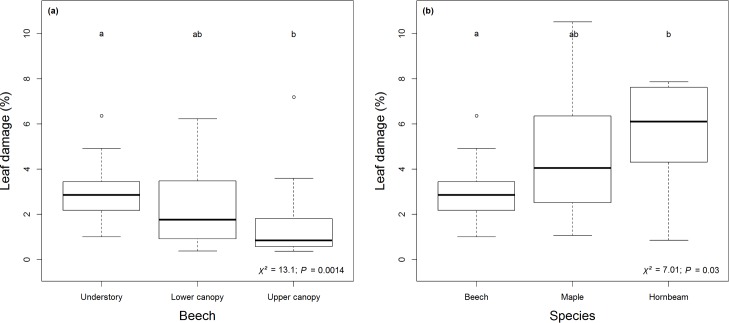
Herbivory patterns of all three species. Leaf damage for (a) *F*. *sylvatica* (n = 60) in different forest layers and (b) tree species (*F*. *sylvatica*: n = 20; *A*. *pseudoplatanus*: n = 14; *C*. *betulus*: n = 10) within the understory. Boxplots with lowercase letters indicate significant differences using Kruskal-Wallis and post-hoc test (*P* < 0.05; df = 2).

Overall, leaf damage of *F*. *sylvatica*, *A*. *pseudoplatanus* and *C*. *betulus* due to herbivore insects showed low values between 1.4% and 5.5% on average. For *F*. *sylvatica*, leaf damage differed between forest layers and showed highest values in the understory (Fig 4A). Differences were significant between upper canopy (1.5% ± 1.6 SD) and understory (2.9% ± 1.3 SD). Within the understory, leaf damage varied between the three species with significant differences between *F*. *sylvatica* (2.9% ± 1.3 SD) and *C*. *betulus* (5.5% ± 2.5 SD) (Fig 4B). *Acer pseudoplatanus* showed highest variation of herbivory (4.6% ± 2.8 SD) and ranged between *F*. *sylvatica* and *C*. *betulus* with no significant differences to them.

**Fig 5:**
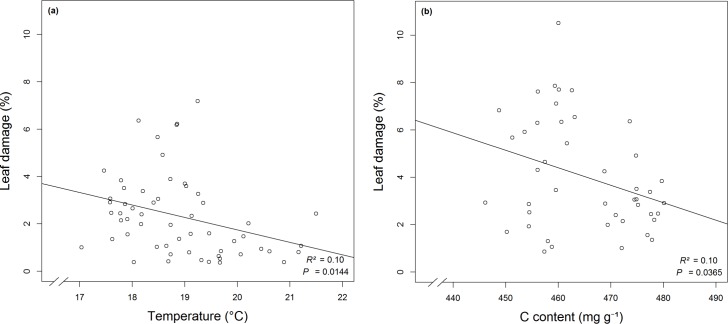
Linear regressions for herbivory levels with selected predictors from model comparison. Leaf damage for *F*. *sylvatica* (a) according to temperature along the vertical forest gradient (n = 57) and (b) based on leaf carbon content for *F*. *sylvatica*, *A*. *pseudoplatanus* and *C*. *betulus* in the understory (n = 44).

Based on the BIC, herbivory of *F*. *sylvatica* across forest layers was slightly better explained by temperature than by humidity or leaf traits (S2 Appendix). Herbivory decreased with increasing temperature from the understory to upper canopies. Within the understory, herbivory of juvenile individuals was better explained by tree species, followed by leaf carbon content as the second best model (S3 Appendix). Linear regressions based on the selected predictors for herbivory of *F*. *sylvatica* (Fig 5A) and the juvenile tree individuals (Fig 5B) showed significant relations.

## Discussion

### Leaf traits along the vertical forest gradient

All measured leaf traits of *F*. *sylvatica*, except chlorophyll content, changed significantly along our vertical forest gradient. Leaves in upper canopies showed lower SLA and leaf nitrogen content as well as higher leaf carbon content and CN ratio than in the understory. The strongest difference was observed in SLA, where the values dropped by half from understory to upper canopy. In general, high temperature leads to an increase in SLA, but it strongly depends on higher soil moisture content [51] and lower CO_2_-concentration [52]. Photosynthesizing leaves in the understory experience higher CO_2_-concentrations (a result of plant and soil respiration) than leaves of upper canopies [53–55], suggesting lower SLA in the understory. However, air humidity also plays an important role for leaf trait characteristics, e.g. leaf length [56]. Our pattern of decreasing SLA from understory to upper canopy coincides with other studies [31,53,57]. The formation of thinner and larger leaf lamina (high SLA values) is a common response to a humid environment [58–60]. This finding supports a stronger influence of humidity than temperature on the characteristic of SLA.

Nutritional quality of plant material with higher nitrogen concentrations is usually increased in sun-exposed leaves [39,61]. However, leaf nitrogen concentration of *F*. *crenata* decreases as light availability increases [41]. We found a similar pattern for *F*. *sylvatica* with lower nitrogen concentration in upper canopy leaves compared to the understory. Since *F*. *sylvatica* is a shade-tolerant tree species, higher leaf nitrogen concentration in lower light environment is a strategy of nitrogen partitioning for more efficient light harvesting [62]. Therefore, higher nutritional quality leaves of *F*. *sylvatica* are found in the understory.

Generally, sun leaves of slow growing species produce more carbon-based secondary defense compounds than shade leaves [33,34]. Chemical defenses of woody plants also vary by growth stage, increasing from juveniles to adults [63]. And, according to the CN balance hypothesis [64], an increase in CN ratio positively correlates with levels of defense compounds. Our pattern of leaf carbon content along the vertical forest gradient is plausible, because concentrations were highest in sun-exposed leaves of adult *F*. *sylvatica* individuals. Furthermore, increased leaf carbon content and CN ratio in leaves of upper canopies of *F*. *sylvatica* suggest higher amounts of carbon based defense compounds.

In our study, a trend of positive correlation between chlorophyll and leaf nitrogen content was visible. Chlorophyll content is certainly dependent on light conditions through its regulating influence on photosynthesis. Studies have presented contrary results with sun leaves of *F*. *sylvatica* showing higher chlorophyll content than shade leaves [65], while in other tree species shade leaves contain more chlorophyll than sun leaves [66]. Measurements of chlorophyll are easy to conduct and a non-destructive method is possible. But since the amount of chlorophyll does not account for the total leaf nitrogen content, it appears to be a weak parameter for herbivory levels.

Growing in the same microclimate, SLA, leaf nitrogen content, CN ratio and chlorophyll content did not differ significantly between the three tree species in the understory. Only leaf carbon content was significantly higher for *F*. *sylvatica* compared to *A*. *pseudoplatanus* and *C*. *betulus*. A defense index based on leaf size, chemical and mechanical defense for large mammalian herbivores rates all our studied tree species with no defensive traits [67]. However, it is known that *F*. *sylvatica* trees typically have the highest amount of phenols compared to co-occurring species in mixed beech forests [68]. Therefore, increased leaf carbon content of *F*. *sylvatica* suggests higher amounts of phenolic defense compounds against herbivore insects compared to *A*. *pseudoplatanus* and *C*. *betulus*, in line with the lower herbivory observed on juvenile *F*. *sylvatica*.

### Forest-layer and species-specific herbivory

Arthropod herbivory is considered to be generally low in temperate forest tree canopies (i.e. up to 7.5% of leaf area eaten), except in outbreak situations [69,70]. Leaf area loss to insects accumulates over time, although the highest damage rates occur on young, high-quality leaves. Folivory rates decline as nutritional quality like nitrogen content decreases and leaf toughness increases in mature foliage [25,27,28,64]. Also, percentages of leaf area removed from lower canopies are significantly greater compared to upper canopies [71]. Typical leaf damage is determined about 6% for *F*. *sylvatica* in late summer [72]. Herbivory levels of our study were somewhat below this range for *F*. *sylvatica* at all forest layers. Highest rates (2.9% ± 1.3 SD) occurred in the understory and where significantly lower than herbivory levels of *C*. *betulus* and *A*. *pseudoplatanus*. Greater herbivory on *A*. *pseudoplatanus*, with leaf area loss reaching 7.6% [73], compared to *F*. *sylvatica* is known from other studies, too [74]. Highest rates of consumed leaf area show similar values around 3.3% for *F*. *crenata* in spring and a further increase in loss of leaf area occurs in lower parts of the canopy after June [41]. Since our field work was done in June, herbivory levels in lower canopies and the understory might have still increased towards the end of the growing season.

### Indirect effects interfere with temperature influence

According to model comparisons, temperature was the best predictor for insect herbivory across forest layers in our study. However, the direction was opposite to the generally known positive relationship between herbivory and temperature: lowest levels of herbivory were observed in upper canopies, where temperatures were highest. Thus, the effect of temperature must have been indirect, either through reduced humidity or through reduced palatability of leaves in the upper canopy. If reduced herbivory in upper canopies is related to higher carbon content, then the mechanism for higher herbivory levels on juvenile *A*. *pseudoplatanus* and *C*. *betulus* compared to *F*. *sylvatica* could be the same as for the vertical forest gradient of herbivory. However, further study is needed to differentiate between air humidity, SLA and leaf chemical composition.

Leaves in upper canopies of *F*. *sylvatica* were tougher and showed lower leaf nitrogen content as well as higher leaf carbon content and CN ratio than in the understory. This pattern is linked to increasing light conditions [41]. There is a strong relationship between light and leaf quality that determines herbivory levels [75,76]. Habitat conditions (microclimate and light) influence leaf traits like foliage lifespan, SLA, and nutrient concentrations [77,78], which are relevant to plant-insect interactions [79,80]. Specific leaf area increases in response to shading [81] because light affects leaf thickness [82]. Furthermore, light increases or decreases leaf nitrogen content depending on plant species [39,41] and increases carbon based defense compounds of leaves [34,83,84]. Example linkages between habitat conditions (microclimate and light) with leaf traits are found in the literature and in our correlations of microclimate (temperature and humidity) with SLA and leaf carbon content. Based on these findings, regulation of our studied leaf traits by given habitat conditions is supported.

**Fig 6:**
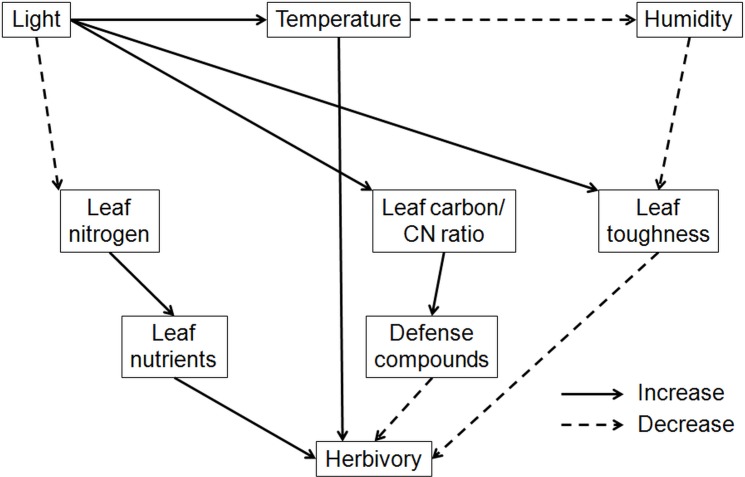
Effects of temperature determining herbivory through direct and indirect pathways and the influence of light on leaf traits along the vertical forest gradient. Data are based on our research and other studies [24,34,39,41,81,83,84].

Since light and temperature are generally expected to be strongly correlated, direct effects of light and temperature on herbivory are likely to be confounded [29]. Light is certainly the most obvious environmental factor which changes along the vertical forest gradient. It is well known that light is reduced from the outer canopy until the forest floor as a result to an increasing tree and shrub cover. Light intensity from upper canopy to the forest floor decreases by 20 times [39]. Therefore, outer canopies of trees experience high irradiances whereas the understory of forests is characterized by low light [32]. The light regime directly affects plants by changes of leaf traits (e.g. formation of sun and shade leaves), growth and physiology (e.g. transpiration and nutrient uptake). Microclimate is also directly influenced by light, creating higher temperatures at the outer canopies and lower temperatures (with less fluctuation) in the understory. Eventually, herbivory is indirectly affected by light due to leaf based changes of host plants or changes in microclimate. Light based variation in leaf nutritional quality and defense compounds might for example account for the suppression of expected high rates of herbivory in warmer habitats [29]. The exact role of light for measured leaf traits and herbivory levels remains unclear because it has not been evaluated in our study. Nonetheless, the effect of temperature on herbivory in our study probably represents indirect effects based on the light regime along the vertical forest gradient. The involved direct and indirect pathways are summarized in Fig 6.

Carbon content increases with light and lowers levels of herbivory presumably due to higher amounts of carbon based defense compounds. For *Fagus* species, light also decreases nutritional quality (nitrogen content) of leaves, which can lower herbivory levels. Higher temperatures in upper canopies go along with decreasing humidity, which lowers SLA and causes tougher leaves that are less palatable for herbivore insects. In our study, low SLA and leaf nitrogen content as well as high leaf carbon content of upper canopy leaves of *F*. *sylvatica* seem to account for reduced herbivory levels. Overall, we believe that the general positive influence of temperature on herbivory in upper canopies is suppressed due to these unfavorable leaf trait changes for herbivore insects.

Due to repetition across a wide geographical range (140 km transect) of ten different forest sites, our results are representative for a large area. Leaf-chewing herbivory can be extremely variable on small spatial scales between individual leaves and branches [42]. Our study shows that a clear overall pattern emerges where hundreds of leaves were pooled on larger scales between individual canopies and among geographically different sites. Therefore, differences of herbivory levels in our study design reflect variation of habitat conditions along the vertical forest gradient and its influences on leaf traits avoiding small-scale patterns between individual leaves and branches.

Overall, the pattern both along the vertical gradient on *F*. *sylvatica* and between three tree species in the understory is in accordance with high carbon content (likely in the form of phenols) limiting herbivory. However, more detailed studies are needed to confirm this mechanism. The same mechanism is expected to underlie a decrease in herbivory with leaf age (seasonal patterns of leaf traits), which was not investigated in the current study.

## Supporting Information

S1 Table Overview of the study sites with elevational and climatic information. (PDF)

S1 Fig Sampling trees of adult and juvenile tree individuals with different potential spots in the understory and forest canopy. (PDF)

S1 Appendix Correlation test and pair plot for all variables. (PDF)

S2 Appendix Model comparison for effects of microclimate and leaf traits on herbivory of *Fagus sylvatica* across forest layers. (PDF)

S3 Appendix Model comparison for effects of tree species and leaf traits on herbivory of *Fagus sylvatica*, *Acer pseudoplatanus* and *Carpinus betulus* in the understory. (PDF)
