# Supplementary material for: Reading the Leaves’ Palm: Leaf Traits and Herbivory along the Microclimatic Gradient of Forest Layers
Source: PLoS One. 2017 Jan 18;12(1):e0169741. doi: 10.1371/journal.pone.0169741 (PMC5242534; doi:10.1371/journal.pone.0169741)
Supplement: S1 Table — (PDF) [file pone.0169741.s001.pdf]

S1 Table. Overview of the study sites with elevational and climatic information.

#	Site	Coordinates	Altitude (m.a.s.l.)	Meteorological station	Precipitation (mm yr^{-1}) 1961-90	1971-2000	2001-12	2012	Temperature (mean yr^{-1}) 1961-90	1971-2000	2001-12	2012
1	Winkelberg	10°24' 51°31'	365	Herzberg	874	870	669	767	n.a.	n.a.	n.a.	8.9
2	Tiefentals Ebene	09°26' 51°39'	271	Wahlsburg	783	811	806	734	8.7	8.7	9.4	9.3
3	Klingenberg/Vaaker Berg	09°38' 51°32'	187	Wahlsburg	783	811	806	734	8.7	8.7	9.4	9.3
4	Schieferstein	10°04' 51°07'	444	Sontra	743	747	698	685	7.4	n.a.	8.8	8.7
5	Heiligenberg	10°01' 51°16'	311	Eschwege	676	659	603	560	8.6	8.9	9.6	9.5
6	Bocksbühl	09°58' 51°25'	368	Göttingen	645	628	637	624	8.7	9.0	9.3	9.4
7	Hubenberg	10°28' 51°26'	376	Friedrichsthal	631	642	637	637	n.a.	n.a.	n.a.	n.a.
8	Feuerkuppe	10°45' 51°23'	309	Sondershausen	542	531	642	n.a.	n.a.	n.a.	9,3	n.a.
9	Heidelberg	10°57' 51°19'	257	Günserode	534	530	535	535	n.a.	n.a.	n.a.	n.a.
10	Eichleite	11°19' 51°16'	143	Artern	474	461	501	462	8.5	8.9	9.6	9.6
