# Supplementary material for: Reading the Leaves’ Palm: Leaf Traits and Herbivory along the Microclimatic Gradient of Forest Layers
Source: PLoS One. 2017 Jan 18;12(1):e0169741. doi: 10.1371/journal.pone.0169741 (PMC5242534; doi:10.1371/journal.pone.0169741)
Supplement: S1 Fig — (PDF) [file pone.0169741.s002.pdf]

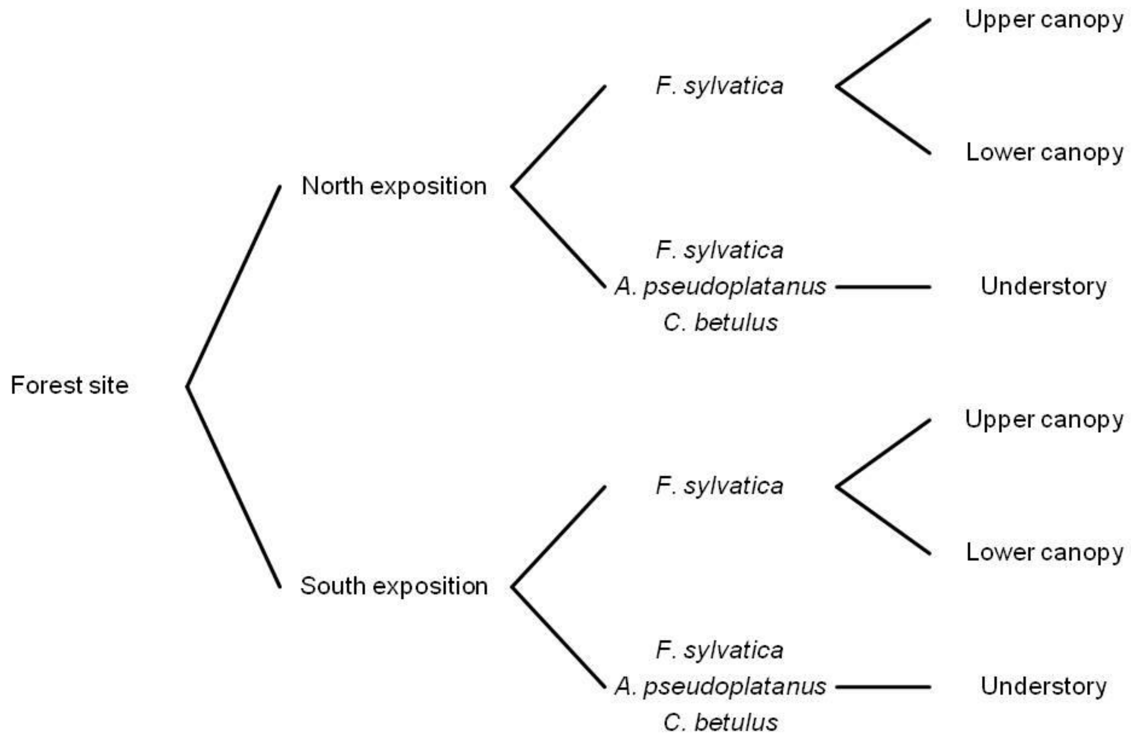

S2 Fig. Sampling trees of adult and juvenile tree individuals with different potential spots in the understory and forest canopy.
