# Supplementary material for: Reading the Leaves’ Palm: Leaf Traits and Herbivory along the Microclimatic Gradient of Forest Layers
Source: PLoS One. 2017 Jan 18;12(1):e0169741. doi: 10.1371/journal.pone.0169741 (PMC5242534; doi:10.1371/journal.pone.0169741)
Supplement: S1 Appendix — (PDF) [file pone.0169741.s003.pdf]

S3 Appendix. Correlation test and pair plot for all variables.

Table. Results of Spearman's correlation test for all variables. Leaf trait parameters are based on data from *F. sylvatica*. Presented rho-values are given with significances (***: *P* > 0.001; **: *P* > 0.01; *: *P* > 0.05).

**Variables**	humid	temp	Ctotal	Ntotal	CN	SLA	chloro
Humid	-	-0.867***	-0.653***	0.447***	-0.496***	0.888***	-0.035
Temp	-0.867***	-	0.590***	-0.335*	0.383**	-0.776***	-0.110
Ctotal	-0.653***	0.590***	-	-0,302*	0.387**	-0.696***	0.128
Ntotal	0.447***	-0.335*	-0,302*	-	-0.992***	0.548***	0.262*
CN	-0.496***	0.383**	0.387**	-0.992***	-	-0.605***	-0.232
SLA	0.888***	-0.776***	-0.696***	0.548***	-0.605***	-	-0.019
Chloro	-0.035	-0.110	0.128	0.262*	-0.232	-0.019	-

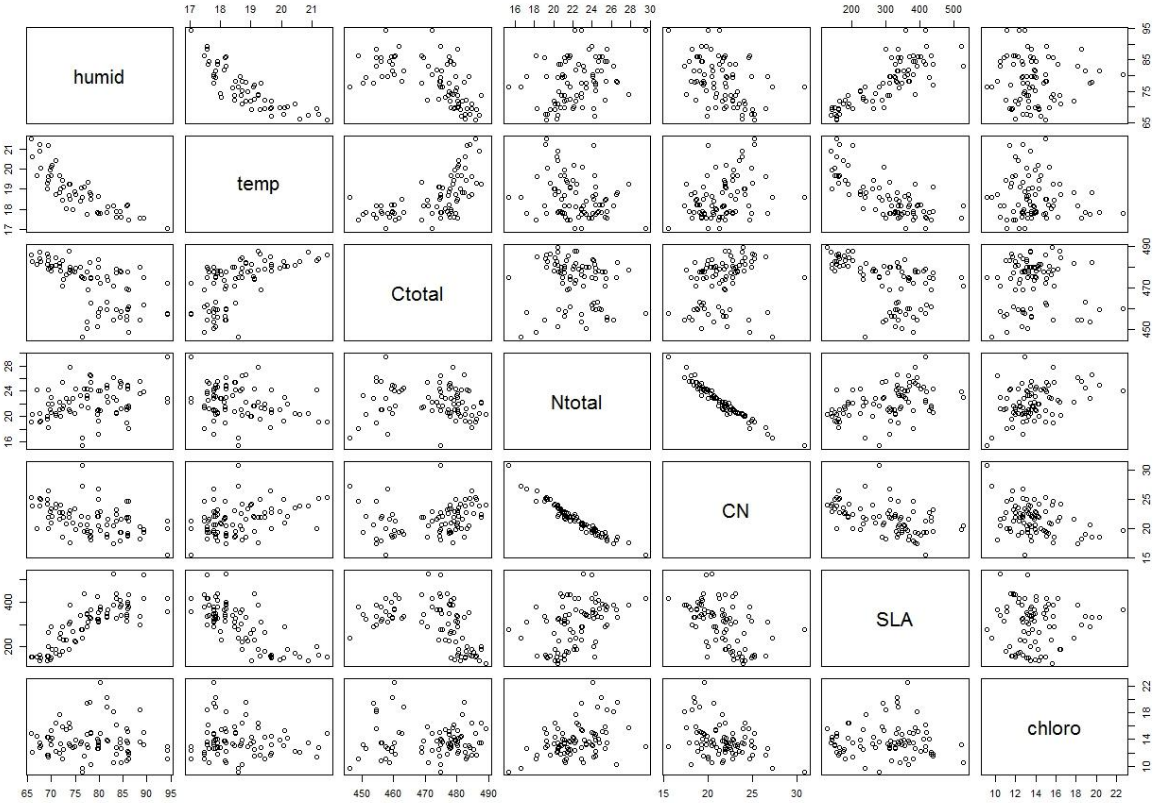

Fig. Pair plot of all variables (humid: n = 57; temp: n = 57; Ctotal: n = 60; Ntotal: n = 60; CN: n = 60; SLA: n = 60; chloro: n = 60) along the vertical forest gradient. Leaf trait parameters are based on data from *F. sylvatica*.
