# Supplementary material for: Reading the Leaves’ Palm: Leaf Traits and Herbivory along the Microclimatic Gradient of Forest Layers
Source: PLoS One. 2017 Jan 18;12(1):e0169741. doi: 10.1371/journal.pone.0169741 (PMC5242534; doi:10.1371/journal.pone.0169741)
Supplement: S2 Appendix — (PDF) [file pone.0169741.s004.pdf]

S4 Appendix. Model comparison for effects of microclimate and leaf traits on herbivory of *Fagus sylvatica* across forest layers. Displayed are the twenty best models according to the Bayesian Information Criterion (BIC). Calculations were done using the R libraries lme4 (Bates et al. 2012) and MuMIn (Barton 2012).

```
Global model call: lmer(formula = sqrt(herb_1) ~ layer + temp_dayav + humid_dayav + Ctotal + Ntotal + chloro + CN + (1 | site), REML = FALSE)
```

Model selection table

	(Int)	chl	CN	Ctt	hmd_dyv	lyr	Ntt	tmp_dyv	df	logLik	BIC	delta	weight
65	0.518900							-0.0199200	4	90.472	-164.8	0.00	0.130
9	-0.083760				2.984e-03				4	90.094	-164.0	0.76	0.089
10	-0.196300	0.008358			2.968e-03				5	92.114	-164.0	0.76	0.089
66	0.405800	0.006705						-0.0187600	5	91.914	-163.6	1.16	0.073
17	0.167700					+			5	91.908	-163.6	1.17	0.072
18	0.072070	0.007142				+			6	93.422	-162.6	2.19	0.043
12	-0.354700	0.010100	0.0041340		3.549e-03				6	92.955	-161.7	3.12	0.027
73	0.308600				1.155e-03			-0.0134300	5	90.644	-161.1	3.70	0.020
67	0.511000		0.0014750					-0.0212200	5	90.587	-161.0	3.81	0.019
44	-1.435000	0.010150	0.0274400		3.880e-03		0.0245200		7	94.575	-160.8	3.92	0.018
69	0.390200			3.024e-04				-0.0207600	5	90.486	-160.8	4.02	0.017
97	0.528100						-0.0002537	-0.0201100	5	90.476	-160.7	4.04	0.017
42	-0.175600	0.009478			3.299e-03		-0.0027500		6	92.468	-160.7	4.10	0.017
68	0.369300	0.007798	0.0032640					-0.0214000	6	92.437	-160.6	4.16	0.016
21	-0.792800			2.023e-03		+			6	92.421	-160.6	4.19	0.016
74	0.063730	0.007669			1.816e-03			-0.0086360	6	92.376	-160.5	4.28	0.015
11	-0.135000		0.0015630		3.207e-03				5	90.224	-160.2	4.54	0.013
14	0.002634	0.008418		-3.914e-04	2.804e-03				6	92.137	-160.0	4.76	0.012
41	-0.079230				3.027e-03		-0.0003526		5	90.100	-160.0	4.79	0.012
13	-0.083800			7.938e-08	2.984e-03				5	90.094	-160.0	4.80	0.012

Random terms (all models): '1 | site'

R code:

```
fullmod<-lmer(sqrt(herb_1)~layer+temp_dayav+humid_dayav+Ctotal+Ntotal+chloro+CN+(1|site),REML=FALSE)
mod1<-dredge(update(fullmod),rank="BIC")
```

References:

Barton K (2012) MuMIn: Multi-model inference. R package version 1.7.11. http://CRAN.R-project.org/package=MuMIn

Bates D, Maechler M and Bolker B (2012) lme4: Linear mixed-effects models using S4 classes. R package version 0.999999-0. http://CRAN.R-project.org/package=lme4
