# Supplementary material for: Reading the Leaves’ Palm: Leaf Traits and Herbivory along the Microclimatic Gradient of Forest Layers
Source: PLoS One. 2017 Jan 18;12(1):e0169741. doi: 10.1371/journal.pone.0169741 (PMC5242534; doi:10.1371/journal.pone.0169741)
Supplement: S3 Appendix — (PDF) [file pone.0169741.s005.pdf]

S5 Appendix. Model comparison for effects of tree species and leaf traits on herbivory of *Fagus sylvatica*, *Acer pseudoplatanus* and *Carpinus betulus* in the understory. Displayed are the twenty best models according to the Bayesian Information Criterion (BIC). Calculations were done using the R libraries lme4 (Bates et al. 2012) and MuMIn (Barton 2012).

```
Global model call: lmer(formula = sqrt(herb_1) ~ species + temp_dayav + humid_dayav + Ntotal + Ctotal + chloro + CN + SLA + (1 | site), REML =
FALSE)
```

Model selection table

	(Int)	chl	CN	Ctt	hmd_dyv	Ntt	SLA	spc	tmp_dyv	df	logLik	BIC	delta	weight
65	0.167700							+		5	69.194	-119.5	0.00	0.114
5	1.052000			-0.001846						4	66.636	-118.1	1.33	0.059
66	0.109500	0.004349						+		6	70.234	-117.8	1.70	0.049
69	-1.050000			0.002565				+		6	70.207	-117.7	1.76	0.048
193	-0.237600							+	0.02259	6	69.842	-117.0	2.49	0.033
6	0.936700	0.004609		-0.001737						5	67.843	-116.8	2.70	0.030
1	0.193100									3	63.994	-116.6	2.83	0.028
197	-2.004000			0.003387				+	0.03143	7	71.513	-116.5	2.93	0.026
73	0.301900				-1.615e-03			+		6	69.536	-116.4	3.10	0.024
81	0.126800					1.830e-03		+		6	69.402	-116.1	3.37	0.021
194	-0.388300	0.004961						+	0.02729	7	71.273	-116.1	3.41	0.021
97	0.134900						8.449e-05	+		6	69.322	-115.9	3.53	0.020
67	0.186400		-8.646e-04					+		6	69.240	-115.8	3.69	0.018
2	0.120100	0.005229								4	65.411	-115.7	3.78	0.017
77	-1.103000			0.003071	-2.262e-03			+		7	70.916	-115.3	4.12	0.015
21	1.013000			-0.001900		2.834e-03				5	67.086	-115.3	4.22	0.014
70	-0.814900	0.003359		0.001975				+		7	70.792	-115.1	4.37	0.013
83	-0.865700		2.282e-02			2.417e-02		+		7	70.778	-115.1	4.40	0.013
74	0.250800	0.004481			-1.721e-03			+		7	70.698	-114.9	4.56	0.012
7	1.081000		-2.151e-03	-0.001811						5	66.901	-114.9	4.58	0.012

Random terms (all models): ‘1 | site’

R code:

```
fullmod<-lmer(sqrt(herb_1)~species+temp_dayav+humid_dayav+ Ntotal+ Ctotal+chloro+CN+SLA+(1|site),REML=FALSE)
mod1<-dredge(update(fullmod),rank="BIC")
```

References:

Barton K (2012) MuMIn: Multi-model inference. R package version 1.7.11. http://CRAN.R-project.org/package=MuMIn

Bates D, Maechler M and Bolker B (2012) lme4: Linear mixed-effects models using S4 classes. R package version 0.999999-0. http://CRAN.R-project.org/package=lme4
